# Molecular and Electrophysiological Role of Diabetes-Associated Circulating Inflammatory Factors in Cardiac Arrhythmia Remodeling in a Metabolic-Induced Model of Type 2 Diabetic Rat

**DOI:** 10.3390/ijms22136827

**Published:** 2021-06-25

**Authors:** Julian Zayas-Arrabal, Amaia Alquiza, Erkan Tuncay, Belma Turan, Monica Gallego, Oscar Casis

**Affiliations:** 1 Departament of Physiology, Facultad de Farmacia, Universidad del País Vasco UPV/EHU, 01006 Vitoria-Gasteiz, Spain; sinjulian@hotmail.com (J.Z.-A.); amaia.alquiza@ehu.eus (A.A.); monica.gallego@ehu.eus (M.G.); 2 Department of Biophysics, Faculty of Medicine, Ankara University, 06100 Ankara, Turkey; Erkan.Tuncay@medicine.ankara.edu.tr; 3 Department of Biophysics, Faculty of Medicine, Lokman Hekim University, 06510 Ankara, Turkey; Belma.Turan@medicine.ankara.edu.tr

**Keywords:** cytokines, arrhythmia, torsade de pointes, potassium current, insulin resistance

## Abstract

Background: Diabetic patients have prolonged cardiac repolarization and higher risk of arrhythmia. Besides, diabetes activates the innate immune system, resulting in higher levels of plasmatic cytokines, which are described to prolong ventricular repolarization. Methods: We characterize a metabolic model of type 2 diabetes (T2D) with prolonged cardiac repolarization. Sprague-Dawley rats were fed on a high-fat diet (45% Kcal from fat) for 6 weeks, and a low dose of streptozotozin intraperitoneally injected at week 2. Body weight and fasting blood glucose were measured and electrocardiograms of conscious animals were recorded weekly. Plasmatic lipid profile, insulin, cytokines, and arrhythmia susceptibility were determined at the end of the experimental period. Outward K^+^ currents and action potentials were recorded in isolated ventricular myocytes by patch-clamp. Results: T2D animals showed insulin resistance, hyperglycemia, and elevated levels of plasma cholesterol, triglycerides, TNFα, and IL-1b. They also developed bradycardia and prolonged QTc-interval duration that resulted in increased susceptibility to severe ventricular tachycardia under cardiac challenge. Action potential duration (APD) was prolonged in control cardiomyocytes incubated 24 h with plasma isolated from diabetic rats. However, adding TNFα and IL-1b receptor blockers to the serum of diabetic animals prevented the increased APD. Conclusions: The elevation of the circulating levels of TNFα and IL-1b are responsible for impaired ventricular repolarization and higher susceptibility to cardiac arrhythmia in our metabolic model of T2D.

## 1. Introduction

Diabetes affects the electrical function of the heart, making it more prone to develop atrial fibrillation, abnormalities in action potential conduction or repolarization, and arrhythmic sudden death [1,2]. The main feature of the cardiac electrical remodeling is a prolonged ventricular repolarization, measured as a prolongation of the QT interval in the electrocardiogram. This increases the risk of torsade de pointes, a type of arrhythmia that can degenerate into ventricular fibrillation and sudden death [3]. Lengthening of the QT interval duration associates with sudden death in type 1 and type 2 diabetic patients [4,5,6]. At the cellular level, prolonged repolarization is the consequence of reduced expression and activity of cardiac ionic channels, mainly the repolarizing Kv4.3 potassium channels [7,8]. Kv4.3 channels are responsible for the transient outward potassium current, I_to_, active during phase 1 of the cardiac action potential [9].

Although the relation between inflammation and heart failure is known [10,11], the mediators that link the inflammatory system with type 2 diabetes-induced electrical abnormalities are unknown. In sterile conditions, low-grade inflammation can be started by the innate immune mechanisms and be prolonged by both innate and adaptive immunity. In this sense, activation of the innate immune system has been proposed as the main pathophysiological mechanism for developing cardiac electrical remodeling in type 1 diabetes [12]. However, more than 90% of diabetic patients have type 2 diabetes. 

On the other hand, in healthy rat ventricular cardiomyocytes TLR activation inhibited Ito, leading to action potential prolongation and triggered activity [13]. Similarly, cytokines such as TNFα and IL-1b also inhibited the transient outward K^+^ current and prolonged ventricular repolarization [12,14]. Type 1 and type 2 diabetic patients had increased TLR activation and proinflammatory cytokine production [15,16,17] and, when type 1 diabetes was induced to Tlr2^−/−^ and IL-1r^−/−^ mice, animals were more resistant to develop arrhythmias after cardiac challenge than wild-type diabetic mice [12].

Most of the knowledge on diabetic cardiac electrical remodeling, from ECGs to behavior of ionic currents, derives from type 1 diabetic animals [7,18,19,20]. Regarding type 2 diabetes, metabolic-induced non-genetic animal models replicate the biochemical characteristics of type 2 diabetes in humans: insulin resistance, moderate hyperglycemia, and hyperlipidemia. These models combine high caloric food that progressively leads to glucose intolerance and insulin resistance, with low-dose STZ to provide the loss of functional beta-cell mass required to establish diabetes [21,22,23,24].

In this work, we aimed to create and characterize a metabolic-induced type 2 diabetic model that developed cardiac electrical remodeling and to identify the key proinflammatory factors involved in the susceptibility to arrhythmia.

## 2. Results

### 2.1. Metabolic Characterization of the Type 2 Diabetic Model

**Figure 1:**
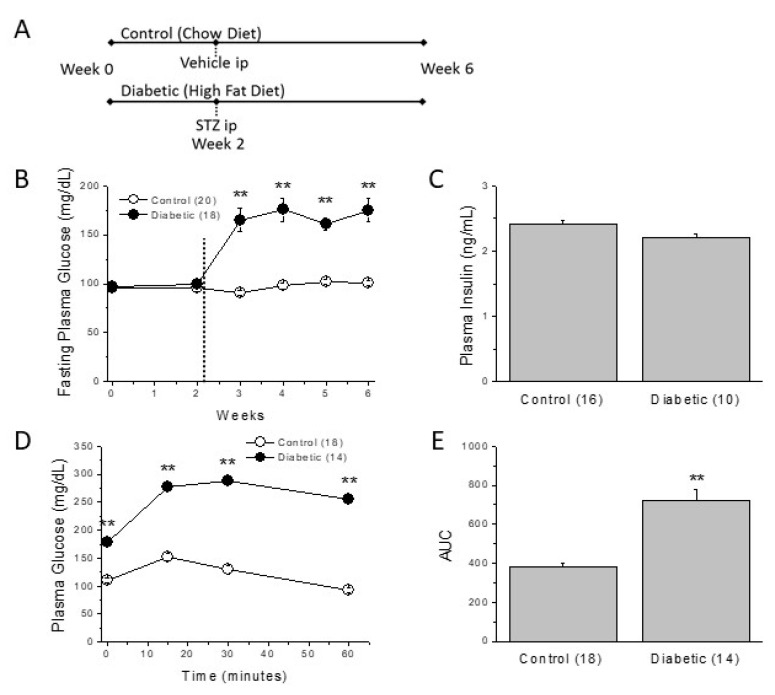
Type 2 diabetic rats show elevated plasma glucose and insulin resistance. (**A**) Description of the experimental protocol for induction of type 2 diabetes. (**B**) Weekly fasting plasma glucose throughout the experimental period. The dotted line indicates the injection of STZ or vehicle. (**C**) Plasma insulin levels after 4 weeks of diabetes. (**D**) IPIGTT performed at the end of the experimental period (week 6). (**E**) The corresponding area under the curve (AUC). In parenthesis (number of animals); ** *p* < 0.01.

The induction of type 2 diabetes begins by feeding of rats with a high-fat diet that progressively led to insulin resistance. Two weeks later, we injected a low-dose STZ (Figure 1A) in order to provide some beta cell loss.

Since the severity of diabetes and associated complications depend on the composition of the diet and the dose of STZ [23], we first confirmed that four weeks were sufficient for the animals to develop the metabolic characteristic of type 2 diabetes. We monitored the fasting plasma glucose (FPG) levels weekly. As can be observed in Figure 1B, after STZ injection FPG levels rapidly rose from 95 mg/dL to approximately 170 mg/dL in the diabetic group (*p* < 0.01). Throughout the experimental period, plasma glucose levels remained elevated and significantly higher than those of the control, chow-fed group. In Chow + STZ and HFD groups, FPG levels were significantly lower than those of the diabetic group. They were also higher than those of the control group, but remained close to the expected physiological limits (Supplementary Table S1).

After four weeks of diabetes, insulin levels were not significantly different between the experimental groups (Figure 1C; Supplementary Table S1). To confirm that the high FPG observed in diabetic animals was due to insulin resistance, we performed an intraperitoneal glucose/insulin tolerance test at the end of the experimental period. After the injection of the bolus of glucose and the insulin, plasma glucose levels were significantly higher in diabetics than in control rats at all the time points tested (Figure 1D,E). In control animals, insulin normalized blood glucose in 30 min. However, diabetic animals could not restore glycemia even after 60 min, indicating the presence of significant insulin resistance. Thus, our model displayed the high plasma glucose and insulin resistance characteristic of type 2 diabetes.

**Figure 2:**
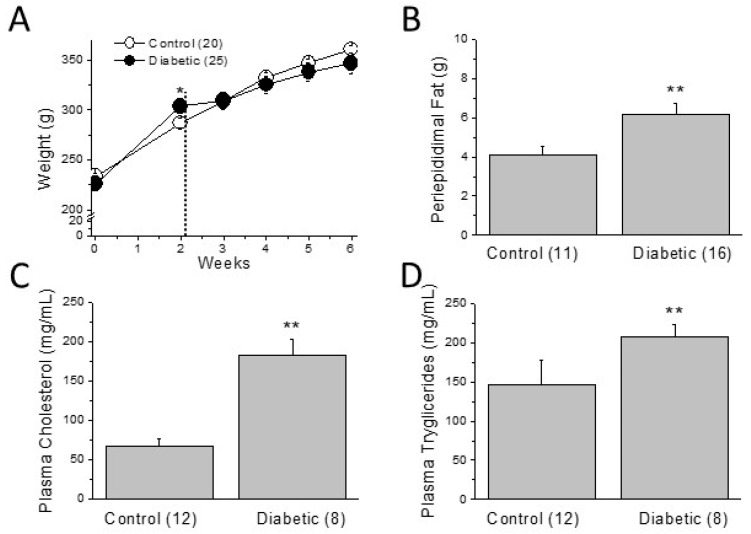
Metabolic disturbances of the type 2 diabetic model. (**A**) Two weeks of HFD increased the body weight, but this effect was reversed by the STZ injection (dotted line). (**B**) Although total body weight is not different between groups at week 6, abdominal fat is significantly greater in diabetic animals. Plasma lipid profile worsened in diabetic animals as can be seen in the increased circulating levels of (**C**) cholesterol and (**D**) triglycerides. In parenthesis (number of animals); * *p* > 0.05; ** *p* < 0.01.

On the other hand, two weeks on a high-fat diet significantly increased the body weight of the diabetic animals, but the STZ injection truncated the tendency (Figure 2A, see weeks 2 to 3). Thus, at the end of the experimental period, body weight was similar in control and diabetic animals. Consistent with that, HFD alone increased the total body weight whereas STZ injection in chow-fed animals reduced the total weight (Supplementary Table S1).

Diabetic animals have similar weight than controls, but have more periepididimal, abdominal fat (Figure 2B). Moreover, they show significant higher levels of plasma triglycerides and cholesterol than those of control animals (Figure 2C,D), indicating a diabetes-induced worsening in plasma lipid profile.

### 2.2. Cardiac Electrical Remodeling in the Type 2 Diabetic Model

**Figure 3:**
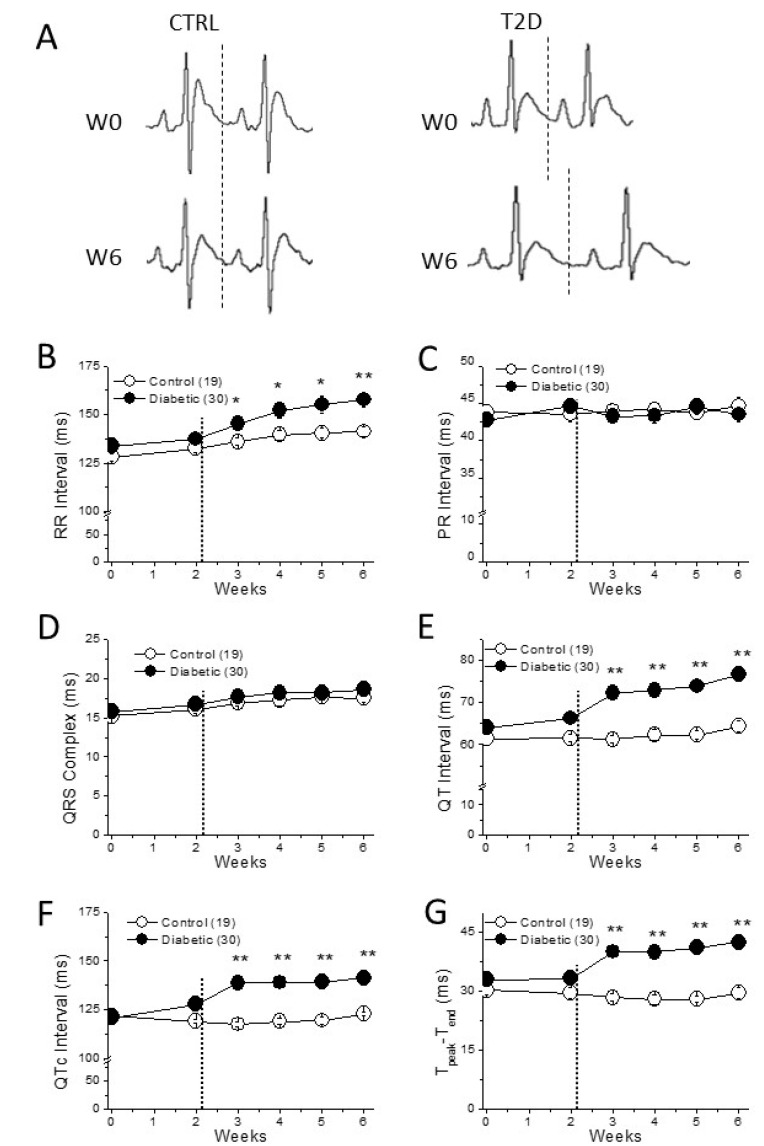
Cardiac electrical remodeling in diabetic heart. (**A**) Electrocardiographic recordings in a control and in a diabetic animal before and after the 6-weeks experimental period. The dotted line shows the end of the T wave. (**B**–**D**) Cardiac impulse generation (RR interval) and conduction (PR interval and QRS complex) throughout the 6 weeks. (**E**,**F**) The main repolarization parameters (QT, QTc, and T_peak-Tend_) are longer in diabetic than in control animals. In parenthesis (number of animals); * *p* < 0.05; ** *p* < 0.01. The dotted line in (**B**–**G**) shows the moment of STZ or vehicle injection.

As expected for a short-term diabetes, no cardiac fibrosis was observed (supplemental Figure S1). Throughout the experimental period, we recorded ECGs (Figure 3A) and analyzed the cardiac electrical characteristics of the diabetic animals. The ECGs of the diabetic animals and age-matched controls showed clear differences. From the first week after STZ injection, RR-interval duration in diabetic rats was significantly longer than that of controls. Thus, as expected, diabetic animals had a lower heart rate compared to that of controls (Figure 3B). The PR-interval duration showed that cardiac impulse conduction at the atrioventricular node was not affected by diabetes (Figure 3C; Supplementary Table S2). Diabetes did not modify either atrial or ventricular depolarization, measured as P wave and QRS complex duration respectively (Figure 3D; Supplementary Table S2). However, durations of QT-interval, QTc (QT interval corrected to the heart rate) and T_peak-_T_end_ were longer in diabetic animals than in controls (Figure 3E–G), confirming that cardiac repolarization is severely affected by diabetes. Last, although the RR-duration in the Chow + STZ group was longer than that in the control group, no significant effect on QTc duration was observed (Supplementary Table S2). 

**Figure 4:**
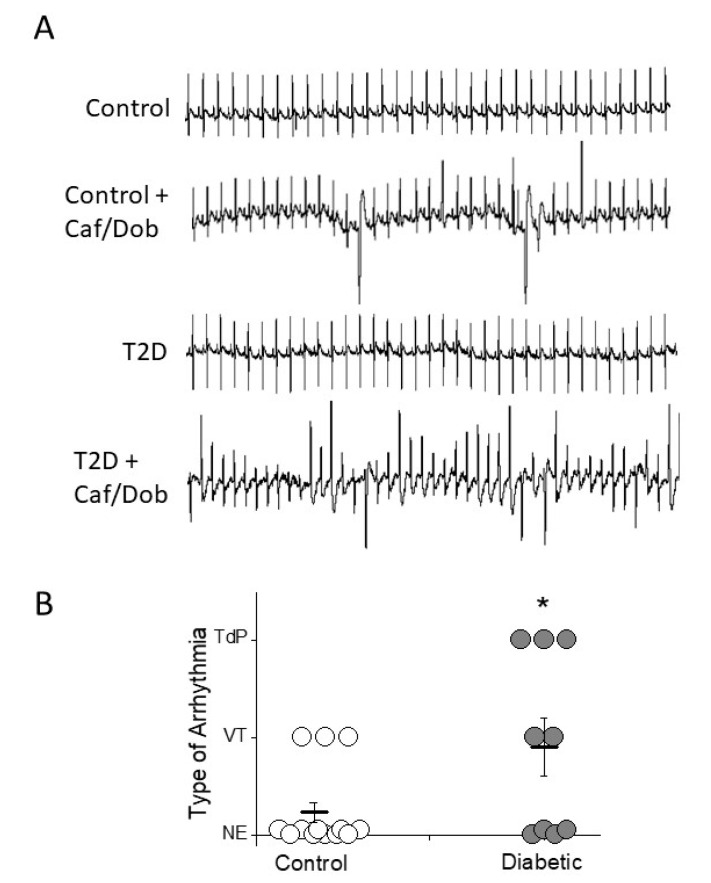
Increased arrhythmia susceptibility in diabetic heart. (**A**) Examples of non-arrhythmic and arrhythmic electrocardiographic recordings in control and diabetic animals after caffeine/dobutamine challenge (Caf/Dob). The ECG of the control animal displays only ventricular premature beats and salvo, whereas the diabetic animal shows episodes of torsade de pointes (TdP). (**B**) Incidence and severity of ventricular tachycardias and TdP in control and diabetic animals. Each arrhythmia was scored as follows: No events (NE) = 0; ventricular tachycardia (VT) = 1; and TdP = 2. The incidence and severity of ventricular arrhythmias after cardiac challenge are higher in diabetic animals than in controls. * *p* < 0.05.

Prolonged QTc-interval and T_peak-_T_end_ durations are arrhythmia surrogates, so we next tested the arrhythmia susceptibility of control and diabetic animals under caffeine/dobutamine challenge (Figure 4A). Ventricular tachyarrhythmias were more frequent in type 2 diabetes than in control animals (55% vs 23%). Moreover, arrhythmia in the diabetic group were more severe than in the control group, since 33% of diabetic animals and 0% of controls developed the life-threatening arrhythmia, torsade de pointes (Figure 4B).

### 2.3. Mechanisms of the Diabetes-Induced Electrical Remodeling

**Figure 5:**
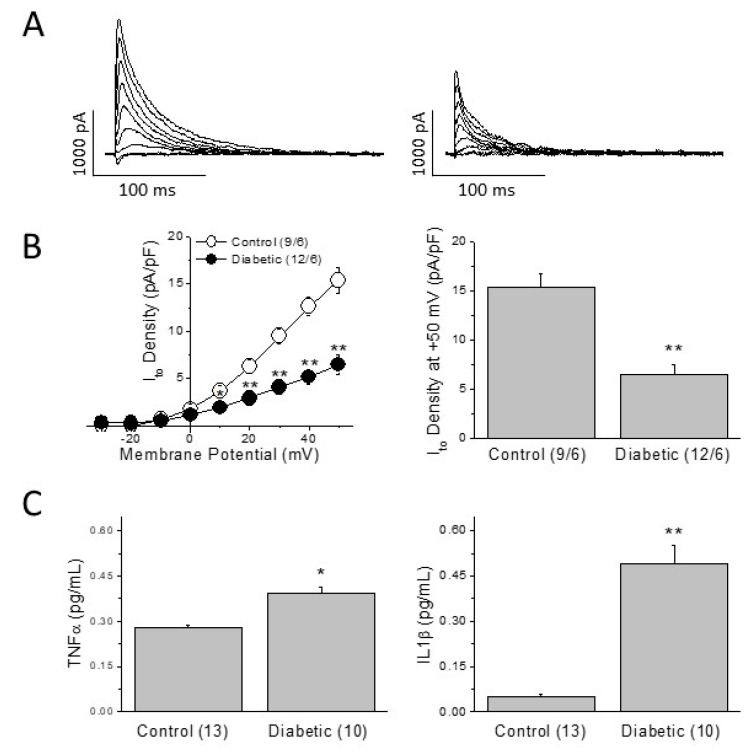
Repolarization capacity and inflammation. (**A**) Current traces of a current-voltage protocol of the transient outward current elicited in a control cell and a diabetic ventricular cell. (**B**) I_to_ density/voltage relationships and I_to_ density at +50 mV in control and diabetic myocytes. In parenthesis (number of cells/number of animals). (**C**) TNFα and IL-1b can reduce the density of I_to_. Plasma levels of TNFα and IL-1b are higher in type 2 diabetic animals than in age-matched controls. Data are mean ± SEM; in parenthesis (number of animals); * *p* < 0.05; ** *p* < 0.01.

In several animal models of diabetes, reduction of the transient outward K^+^-current (I_to_) has been proposed as a main cause of prolonged repolarization [18,19]. We recorded I_to_ in left ventricular myocytes isolated from control and diabetic animals and confirmed that our metabolic-induced diabetic model inhibited the current amplitude. Figure 5A shows I_to_ recordings in a control and a diabetic cell of similar size. The current traces were elicited by depolarizing pulses ranging from −30 to + 50 mV, from a holding potential of −60 mV. In diabetic cardiomyocytes, I_to_ was significantly smaller than in control cells at positive voltages. At +50 mV, I_to_ density was approximately 50% smaller in diabetic than in control cells (Figure 5B). Therefore, the alteration of the repolarizing current is consistent with the prolonged QTc observed in the ECG.

In type 1 diabetic animals, I_to_ inhibition has been attributed to the diabetic inflammatory status [12]. In that regard, TNFα and IL-1b are circulating pro-inflammatory cytokines known to reduce the functional expression of I_to_. Next, we compared the plasmatic levels of these cytokines in our type 2 diabetic animals and age-matched controls. At the end of the experimental period, diabetic animals had significantly higher levels of TNFα and IL-1b than control animals (Figure 5C).

### 2.4. Role of Circulating Factors on Cardiac Electrical Remodeling 

These previous results suggest that in this type 2 diabetic model, elevation of plasmatic TNFα, and IL-1b levels could cause I_to_ reduction. This would lead to prolonged repolarization and higher susceptibility to arrhythmia under cardiac challenge. To confirm whether diabetes-associated circulating factors could induce cardiac electrical remodeling, we exposed cardiomyocytes isolated from control rats to plasma extracted from diabetic animals and recorded action potentials. 

**Figure 6:**
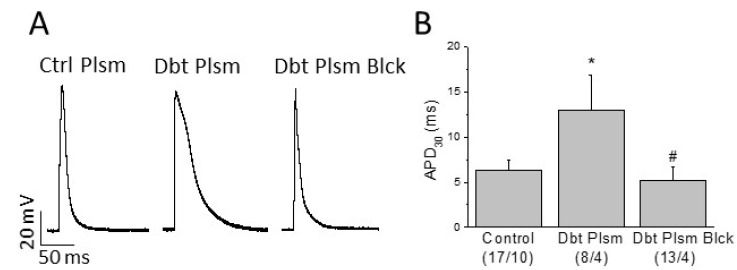
Role of circulating mediators on cardiac electrical remodeling. (**A**) Typical action potentials recorded in myocytes isolated from the right ventricle of healthy animals incubated for 24 h in DMEM supplemented with: plasma from healthy animals (Ctrl Plsm); with plasma from diabetic animals (Dbt Plsm); or plasma extracted from type 2 diabetic animals plus TNFα and IL-1b receptor blockers (Dbt Plsm Blck). (**B**) Incubation with diabetic plasma prolongs the action potential duration at 30% of repolarization (APD_30_), and this effect is prevented by TNFα plus IL-1b receptor blockers. Data are mean ± SEM; in parenthesis (number of cells/number of animals); * *p* < 0.05 with respect to control; # *p* < 0.05 with respect to Dbt Plsm.

Ventricular myocytes isolated from control rats were incubated 24 h in DMEM supplemented with either plasma obtained from non-diabetic animals; or plasma extracted from type 2 diabetic animals, with or without added TNFα (50 μM) and IL-1b (50 μg/mL) receptor blockers (Figure 6 and Table 1).

Resting membrane potential and AP amplitude were similar in the three experimental groups. However, incubation of cardiomyocytes with plasma extracted from diabetic animals significantly prolonged the action potential duration at the 30, 50, and 90% of repolarization (Figure 6 and Table 1). Blockade of the TNFα and IL-1b cytokine receptors prevented the diabetic plasma-induced effect. Therefore, we found that diabetic plasma components altered the electrical behavior of normal cardiomyocytes regarding major pro-arrhythmic parameters. 

## 3. Discussion

In this work, we characterized a type 2 diabetic rat model with cardiac electrical remodeling. Diabetic animals had prolonged QTc, reduced I_to_ and high susceptibility to develop arrhythmia under cardiac challenge. In diabetic animals, we found increased plasmatic levels of TNFα and IL-1b. These circulating proinflammatory factors were responsible for impaired ventricular repolarization that underlies the higher susceptibility to arrhythmia in our metabolic model of type 2 diabetes.

Different combinations of high-caloric diets (fructose rich, fat rich) and low STZ doses have been used to induce type 2 diabetes in mammals [21,22,23]. In this work, we used a diet with 45% Kcal from lipids to cause progressive insulin resistance. After two weeks, we injected a low dose of STZ intraperitoneal to facilitate some beta cell destruction. About 48 h after STZ administration, diabetes was confirmed by elevated FPG and animals continued consuming a high-fat diet. We weekly measured the metabolic and electrocardiographic parameters and found that the expected diabetes-associated changes were well established for one week and maintained four weeks after STZ injection. Thus, in this work, we characterized an animal model with the main metabolic and electrocardiographic characteristics of type 2 diabetic patients in a reasonable period of 6 weeks.

Recent morphological studies show that in the metabolically induced type 2 diabetes model in rats, the pancreas is characterized by a normal exocrine tissue, together with islets with disrupted boundaries, decreased size, and reduced cell number. Islet cells have vacuolated cytoplasm and small-sized deformed nucleus [25,26]. In addition, immunohistochemical staining show that the reduction in cell number is due to a selective loss of β-cells in the damaged pancreatic islets [26,27].

Our experimental model was developed to mimic the clinical features of human type 2 diabetes. Thus, we generated a mild diabetic condition avoiding massive beta cells loss and the consequent development of type 1 diabetes. In this work, the levels of circulating insulin together with peripheral insulin resistance ensure that our experimental model corresponds to type 2 diabetes.

Diabetic animals replicated the classical high-fasting plasma glucose and insulin resistance characteristics of type 2 diabetic patients. Since fasting plasma insulin was similar in diabetics than in control animals, the high blood glucose was due to insulin resistance and the inability of the pancreas to compensate by increasing insulin production. Moreover, as described for type 2 diabetic patients [28], our type 2 diabetic animals had more abdominal fat than the control animals, hypercholesterolemia, and hypertriglyceridemia. Our experimental data are in line with the clinical findings in humans with type 2 diabetes. Correspondingly, the ECGs of diabetic patients show slower heart rate and prolonged duration of the QT-interval, indicating repolarization abnormalities associated with increased risk of ventricular arrhythmias and mortality [2,29]. Indeed, in our present study, diabetic animals, as well as the animals in the Chow + STZ group, had a lower heart rate than control animals measured as longer RR-intervals. However, only animals in the diabetic group (high-fat diet plus STZ) had repolarization abnormalities. Diabetic rats showed the expected prolongation of the repolarization time, seen as the prolonged QT and QTc intervals and the prolonged T_peak-_T_end_, an electrocardiographic marker of the transmural dispersion of repolarization. Prolonged QTc and T_peak-_T_end_ are used in clinical settings to predict the risk of ventricular arrhythmia and sudden cardiac death [30,31,32]. In this sense, when subjected to proarrhythmic conditions diabetic animals developed more arrhythmic episodes and more severe than control animals. It is important to note that cardiac challenge induced the potentially lethal arrhythmia torsade de pointes in one out of three type 2 diabetic animals. By contrast, none of the control animals developed torsade de pointes under the same challenge.

At the cellular level, diabetes can induce lengthening in the cardiac repolarization time by affecting the expression and activity of several cardiac ionic channels and their corresponding ionic currents. In models of type 1 diabetes, prolonged repolarization is caused by a reduction of the transient outward K^+^ current, I_to_ [7,8,18,19]. Our results in the type 2 diabetic model are in agreement with that. We found that I_to_ density in type 2 diabetic animals is smaller than in control animals. The strong reduction on I_to_ density caused by diabetes reduced the repolarization capacity of the ventricular cells. This can explain the prolonged repolarization time of the whole heart observed in the electrocardiogram. 

The accumulation of abdominal fat is recognized as the origin of a generalized low-grade inflammatory status of the whole body, with increased circulating cytokines such as IL-6, TNFα, IFN-γ, TGF-β, MCP-1, or IL-1b [33]. Among them, TNFα and IL-1b are two cytokines known to be upregulated in type 2 diabetic patients [16], which have demonstrated in vitro and in vivo animal models the ability to reduce the functional expression of the cardiac repolarizing current I_to_. TNFα modifies I_to_ inactivation and reduces the channel expression through iNOS and oxidant species [14], whereas IL-1b receptor activation reduces I_to_ expression through NLRP3 activation [12]. Moreover, it has been recently demonstrated the role of both TNFα and IL-1b in the initiation and maintenance of cardiac arrhythmias in humans [34,35]. Here, we found that plasma levels of both cytokines were significantly higher in our type 2 diabetic animals than in their age-matched controls. Therefore, as in diabetic patients, our type 2 diabetic model had an inflammatory component that can affect different organs including the heart.

We confirmed that high plasmatic levels of TNFα and IL-1b cause a cellular electrical remodeling that impairs the ability of cardiac myocytes to repolarize. Incubation of control myocytes with plasma obtained from diabetic animals resulted in a diabetic-like electrical phenotype. Prolonged APD was prevented by preincubating the diabetic plasma with TNFα and IL-1b receptor blockers. Thus, diabetes-associated circulating proinflammatory factors induce electrical remodeling in cardiac cells. As a result, there is an impairment in major pro-arrhythmic parameters, increasing the risk to generate arrhythmia. Although an increase of cardiac macrophages has been described in the diabetic heart [12,36], our results point to a role of systemic over local inflammation on the cardiac electrical remodeling.

Different phase 1 and 2 clinical trials in diabetic patients of recent onset showed that anti-IL-1b agents are safe but are not efficacious as single agents for diabetes control [37,38]. However, our data demonstrate that TNFα and IL-1b receptor blockade prevents the cardiac electrical remodeling in diabetes. Thus, cytokine receptor blockade could arise as an interesting combination therapy with first choice antidiabetics for diabetic patients with cardiovascular risk. 

### 3.1. Limitations

In this study, we used a rat model of type 2 diabetes, to reproduce the cardiac electrical remodeling observed in type 2 diabetic patients. One limitation of the study arises from the use of an animal model. In the human heart, the main repolarizing currents are the transient outward K^+^-current, I_to_, and the rapid delayed rectifier, I_Kr_ [39]. In the rat heart, I_to_ is the most important repolarizing current whereas the I_Kr_ is not relevant [40]. However, using different animal models such as rat, mouse, rabbit, or dog, which express functional I_to_ and/or I_Kr_, it has been consistently described that diabetic cardiomyopathy reduces the functional expression of I_to_, but does not modify the characteristics of I_Kr_ [7,8,20].

Another limitation could be the enormous diversity of circulating cytokines. A recent study [41], described that almost 20 circulating cytokines such as TNFα, IL-1a, IL-1b, GM-CSF, IL-10, IL-12, etc., are elevated in plasma of type 2 diabetic patients. Randeria et al. correlated this inflammatory profile with a hypercoagulable state and vascular dysfunction. Here, we demonstrated the role of two of them, TNFα and IL-1b, in the cardiac electrical remodeling of type 2 diabetes. Thus, further studies are needed to shed light on the circulating levels of other cytokines in diabetes and the effects of this inflammatory profile on the myocardium and other organs.

### 3.2. Summary

We characterized a type 2 diabetes model based on a high-fat diet and STZ that reproduces the cardiac electrical remodeling described in type 2 diabetic patients. We have shown that plasmatic cytokines have a role in the diabetes-induced cardiac repolarization abnormalities that could lead to an increase in the susceptibility to arrhythmia.

## 4. Materials and Methods

### 4.1. Induction of Diabetes and In Vivo Treatments

The investigation fulfilled the Spanish (RD 1201/2005) and European (D2003/65/CE and R2007/526/CE) rules for animal care used for experimental and other research purposes, and has been approved by the Ethics Committee for Animal Care of the Universidad del País Vasco (with references CEBA/111a/2010 and CEBA/273M/2012). Approximately 8-week-old and 200–220 g weight at the beginning of the experiment male Sprague-Dawley rats (from the Animal Facility, University of the Basque Country, Vitoria-Gasteiz, Spain) were used. Animals were housed under a 12:12 h light:dark cycle in a temperature-controlled room (23 °C).

Animals were randomized in two experimental groups (randomization was performed with each batch of animals): control and type 2 diabetic. Type 2 diabetes was induced by the combination of high-fat diet (HFD: 45% Kcal from lipids, 35% Kcal from carbohydrates and 20% Kcal from proteins; Research Diets) to induce insulin resistance and a low-dose streptozotocin (STZ) to provide loss of functional beta cell mass (modified from ref. [22]). The control group received standard chow. At week 2 on HFD, rats received a single intraperitoneal injection of STZ (Sigma-Aldrich Co., St. Louis, MO, USA, 35 mg/kg in 0.1 mol/L citrate buffer, pH 4.5). Approximately 48 h later diabetes was confirmed by fasting blood glucose levels >126 mg/dL in two independent measurements.

On the other hand, two more experimental groups were used as additional controls. The Chow + STZ group consisted of control animals fed with standard chow who received an STZ injection. In the HFD group, control animals were fed with a high-fat diet and had no STZ injection.

### 4.2. Blood Measurements

Animals fasted overnight were anesthetized with isoflurane inhalation and blood from the tail vein was collected in heparinized capillaries. Fasting blood glucose was determined with glucose reagent strips in a standard automated glucometer ContourXT (Menarini Diagnostics, Basel, Switzerland). Plasma insulin, IL-1b, and TNFα were measured by ELISA kits (Merck-Millipore, Darmstadt, Germany; BioRad, Hercules CA, USA) and plasma cholesterol and triglycerides were determined using colorimetric test kits (SPINREACT, Girona, Spain) following the manufacturer instructions.

### 4.3. Combined Intraperitoneal Insulin and Glucose Tolerance Test, IPIGTT

We used a combined intraperitoneal insulin and glucose tolerance test or IPIGTT [22] to avoid severe hypoglycemic episodes. Animals were fasted overnight and then kept under light anesthesia with isofluorane. Glucose (1 g/kg) and insulin (1 U/kg) were injected intraperitoneally. Blood was extracted from the tail vein and blood glucose was measured right before (0 min) and 15, 30, and 60 min after the injection. Data were plotted and the area under the curve (AUC) was calculated with Origin 8.0 software (OriginLab, Northampton MA, USA).

### 4.4. In Vivo ECG Recordings

ECGs were recorded in conscious animals by a noninvasive method. Surgical stainless steel electrodes, placed under the skin in the DII lead, were connected to a Biopac MP35 recording system controlled by the Biopac Pro software (Biopac Systems Inc., Goleta CA, USA). Data were automatically analyzed with the LabChart 7.0 software (AD Instruments, Oxford, UK). Electrocardiographic parameters were calculated as the average of 50–80 beats. Corrected QT interval was calculated using the Fridericia formula: QTc = QT/(RR/1000)^0.33^.

At the end of the experimental period, an arrhythmia susceptibility test was performed in anesthetized animals [42]. Animals received 120 mg/kg of caffeine intraperitoneal (Merck-Millipore, Darmstadt, Germany) and 50 μg/kg of dobutamine intravenous (Merck-Millipore, Darmstadt, Germany). ECG was recorded for 5 min before and for 15 min after the caffeine/dobutamine challenge. The type of arrhythmia was identified according to the Lambeth Convention guidelines [43].

### 4.5. Cardiomyocyte Isolation

Animals were anesthetized with intraperitoneal injections of 50 mg/kg ketamine (Dechra, Northwich, UK) and 7 mg/kg xylazine (Laboratorios Calier, Leon, Spain). Anesthetized animals were weighted and the hearts were removed and perfused via the aorta at 37 °C for 5 min with Tyrode solution containing (final concentrations in mmol/L): NaCl 130, KCl 5.4, NaHCO_3_ 5.8, MgCl_2_ 1.05, CaCl_2_ 1.8, NaH_2_PO_4_ 0.42, dextrose 12, taurine 20, HEPES-Na^+^ 25, adjusted to pH 7.4 with NaOH. Next, samples were switched to a nominally Ca^2+^-free Tyrode solution for 10 min; and then to Ca^2+^-free solution containing 1 mg/mL collagenase type II (Worthington, Lakewood NJ, USA) and 0.03 mg/mL protease Type XIV (Merck-Millipore, Darmstadt, Germany) for 15 min. The enzymes were washed for 5 min with KB solution (in mmol/L): taurine 10, glutamic acid 70, creatine 5, succinic acid 5, dextrose 10, KH_2_PO_4_ 10, KCl 20, HEPES-K^+^ 10, EGTA-K^+^ 0.2, adjusted to pH 7.4 with KOH. Last, the right ventricle was excised and isolated cells were obtained by mechanical agitation.

### 4.6. Patch-Clamp Experiments

Transient outward potassium currents and action potentials were recorded at room temperature (20–22 °C), using the whole-cell configuration of the Patch-Clamp technique with an Axopatch 200B patch-clamp amplifier (Molecular Devices, San Jose CA, USA). Recording pipettes were obtained from borosilicate capillary glass (Sutter Instruments, Novato CA, USA) and had a tip resistance of 1–3 MΩ when filled with the internal solution (final concentrations in mmol/L): L-aspartic acid (potassium salt) 80, KH_2_PO_4_ 10, MgSO_4_ 1, KCl 50, ATP-Na_2_ 3, EGTA-K^+^ 10, HEPES-K^+^ 5, adjusted to pH 7.2 with KOH. Following the patch rupture, whole-cell membrane capacitances were measured from the integration of the capacitive transients elicited by voltage steps from −50 to −60 mV. Series resistances were compensated 80% to minimize errors.

For I_to_ recordings, freshly isolated myocytes were allowed to settle for two hours before the start of the experiments. The external bathing solution for I_to_ recording was (final concentrations in mmol/L): NaCl 86, KCl 4, CaCl_2_ 0.5, MgCl_2_ 1, CoCl_2_ 2, dextrose 11, TEA-Cl^−^ 50, HEPES-Na^+^ 10, adjusted to pH 7.4 with NaOH. In the voltage-clamp configuration of the amplifier, starting from a holding potential of −60 mV, 500 ms depolarizing voltage pulses between −30 and +50 mV were applied at a frequency of 0.1 Hz to allow complete recovery of I_to_ from inactivation. The TEA-resistant, time-independent I_ss_ was digitally subtracted.

In another group of experiments, the freshly isolated left ventricular myocytes were incubated for 24 h in DMEM supplemented with either plasma obtained from control rats; plasma extracted from type 2 diabetic animals; or plasma extracted from type 2 diabetic animals supplemented with TNFα and IL-1b receptor blockers (TNF-α Inhibitor; CAS 1049741-03-8, Merck-Millipore, Darmstadt, Germany; and Anakirna^®^, Kineret, Swedish OrphanBiovitrum, Stockholm, Sweden). Then, the action potentials were recorded in those cells with a bathing solution (final concentrations in mmol/L): NaCl 136, KCl 5.4, MgCl_2_ 1, CaCl_2_ 1.8, dextrose 11, HEPES-Na^+^ 10, adjusted to pH 7.4 with HCl. In the current-clamp configuration of the amplifier, 4 ms current pulses were applied at twice the threshold at a frequency of 2 Hz.

Voltage-clamp and current-clamp experimental protocols were controlled with the Clampex program and all online recording signals were analyzed with the Clampfit program of the pClamp 10.2 software (Molecular Devices, San Jose CA, USA). I_to_ amplitudes were normalized to cell capacitance and expressed as pA/pF.

### 4.7. Statistical Analysis

Data are presented as means ± standard error of the mean (SEM). Data were compared with Student’s *t*-test or analysis of variance followed by Bonferroni’s *t*-test. A *p* value lower than 0.05 was considered as statistically significant.

## Figures and Tables

**Table 1:** Diabetic circulating factors prolongs action potential duration in ventricular myocytes. Effects of control plasma, diabetic plasma, and diabetic plasma with blockers of TNFα and IL1β receptors on ventricular action potential.

	RMP (mV)	APA (mV)	APD90 (ms)	APD50 (ms)	APD30 (ms)
Control (17/10)	−64.0 ± 6.7	72.4 ± 7.8	24.7 ± 4.5	9.0 ± 2.1	6.3 ± 1.2
Diabetic Plasma (8/4)	−63.0 ± 8.2	74.7 ± 8.0	57.4 ± 14.5 *	20.7 ± 5.6 *	13.0 ± 3.9 *
Diabetic Plasma Blocked (13/4)	−64.4 ± 2.0	68.4 ± 4.4	33.3 ± 3.2	8.3 ± 1.9 ^#^	5.2 ± 1.5 ^#^

Resting membrane potential (RMP), action potential amplitude (APA), and action potential duration (APD) at 30, 50, and 90% of repolarization. In parenthesis (number of cells/number of animals); * *p* < 0.05 with respect to control; ^#^ *p* < 0.05 with respect to Dbt Plsm.

## Data Availability

Data is contained within the article or supplementary material.

## Supplementary Materials

The following are available online at https://www.mdpi.com/article/10.3390/ijms22136827/s1.
