# Supplementary material for: Molecular and Electrophysiological Role of Diabetes-Associated Circulating Inflammatory Factors in Cardiac Arrhythmia Remodeling in a Metabolic-Induced Model of Type 2 Diabetic Rat
Source: Int J Mol Sci. 2021 Jun 25;22(13):6827. doi: 10.3390/ijms22136827 (PMC8268936; doi:10.3390/ijms22136827)
Supplement: Supplementary file 1 [file ijms-22-06827-s001.zip › ijms-1245779-supplementary.pdf]

Supplementary Table S1. Animal characteristics for control, chow + streptozotocin, high-fat diet (HFD), and DIABETIC (HFD + STZ) groups, measured at week 6.

	Control	Chow + STZ	HFD	Diabetic
Body weight (g)	364.1 ± 4.2	331.7 ± 12.3 *	374 ± 4.7 *	346.8 ± 10
Glucose (mg/dL)	101.1 ± 2.5	127.8 ± 7.2 **,#	122 ± 5.6 **,#	175.05 ± 12 **
Cholesterol (mg/dL)	67.1 ± 9.4	100 ± 34	198.6 ± 16.8 **	183 ± 20.6 **
Triglycerides (mg/dL)	146.1 ± 32	205.1 ± 60.3	169.2 ± 57	207.7 ± 15.5 *
IPIGTT (AUC)	394 ± 16	413 ± 92	519 ± 107 *	723 ± 56 **
Insulin (ng/mL)	2.2 ± 0.2	1.9 ± 0.01	2.2 ± 0.1	2.4 ± 0.2
Epid. Fat (g)	4.1 ± 0.4	3.3 ± 0.4 *,##	7.2 ± 0.5 **	6.2 ± 0.5 **

Data are mean ± SEM, n = 4-17 per group; * $p < 0.05$, ** $p < 0.01$ compared to control; # $p < 0.05$,
$p < 0.01$ compared to DIAB group.

Supplementary Table S2. Electrocardiographic characteristics for control, chow + streptozotocin (Chow + STZ), high-fat diet (HFD), and DIABETIC (HFD + STZ) groups, measured at week 6.

	Control	Chow + STZ	HFD	Diabetic
RR (ms)	141.2 ± 2.5	159.6 ± 7.24 **	136.2 ± 1.17	157.6 ± 3.9 **
PR (ms)	44.48 ± 1.01	44.41 ± 1.36	45.34 ± 1.16	44.07 ± 0.92
P (ms)	13.65 ± 0.35	14.68 ± 0.82	15 ± 0.58	14.94 ± 0.42
QRS (ms)	17.16 ± 0.3	17.04 ± 0.71	16.32 ± 0.82	18.79 ± 0.93
QT (ms)	64.2 ± 1.3	66 ± 2.2	67.5 ± 1.5	76.5 ± 1.5 **
QTc (ms)	122.8 ± 1	125.5 ± 1.56	116.6 ± 1.2	141.4 ±1.3 **
T_{peak}-T_{end} (ms)	29.4 ± 1.4	33.9 ± 1.1	35 ± 1.5	42.4 ± 1.3 **

Data are mean ± SEM, n = 6-30 per group; ** $p < 0.01$ compared to control.

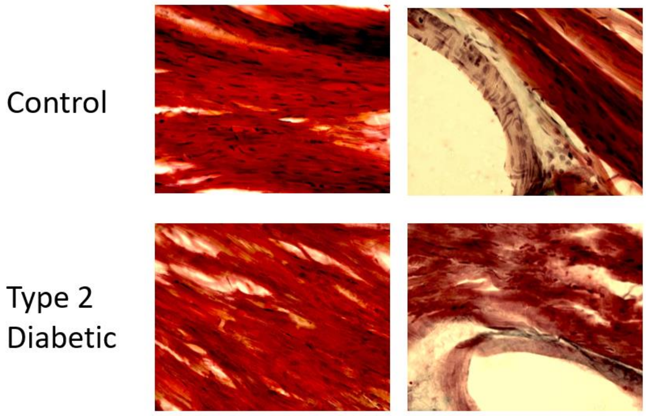

Supplementary Figure 1. Myocardial structural characteristics. Masson's trichrome stains show the absence of either interstitial or perivascular fibrosis in the type 2 diabetic hearts compared to controls.
